# Association of environmental enteropathy with prediabetes and diabetes: A cross-sectional study among Tanzanian adults

**DOI:** 10.1371/journal.pone.0327166

**Published:** 2025-07-03

**Authors:** George PrayGod, Belinda Kweka, Evangelista Malindisa, Andrea M. Rehman, Ruth Frikke-Schmidt, Christina Christoffersen, Rikke Krogh-Madsen, Daniel Faurholt-Jepsen, Henrik Friis, Paul Kelly, Suzanne Filteau

**Affiliations:** 1 Muhimbili Research Centre, National Institute for Medical Research, Dar es Salaam, Tanzania; 2 Mwanza Research Centre, National Institute for Medical Research, Mwanza, Tanzania; 3 Department of Physiology, Catholic University of Health and Allied Sciences, Mwanza, Tanzania; 4 Faculty of Epidemiology and Population Health, London School of Hygiene & Tropical Medicine, London, United Kingdom; 5 Department of Clinical Medicine, University of Copenhagen, Denmark; 6 Department of Clinical Biochemistry, Rigshospitalet, Copenhagen, Denmark; 7 Centre of Inflammation and Metabolism and Centre for Physical Activity Research, Rigshospitalet, University of Copenhagen, Denmark; 8 Department of Infectious Diseases, Copenhagen University Hospital Hvidovre, Denmark; 9 Department of Infectious Diseases, Rigshospitalet, Denmark; 10 Department of Nutrition, Exercise and Sports, University of Copenhagen, Denmark; 11 University Teaching Hospital, Lusaka, Zambia; 12 Faculty of Medicine and Dentistry, Queen Mary University of London, London, United Kingdom; Livingstone Center for Prevention and Translational Science, ZAMBIA

## Abstract

**Objectives:**

Environmental enteropathy (EE) may increase the risk of diabetes, but data are limited. We assessed the role of EE on markers of glucose metabolism.

**Methods:**

Cross-sectional study among Tanzanian adults assessing EE and diabetes was conducted between 2019 and 2021. Data on demography, body mass index (BMI), Human immunodeficiency virus (HIV), EE (i.e., fecal myeloperoxidase, lipopolysaccharide binding protein, and markers of intestinal permeability and absorption capacity), glucose and insulin were collected. Data reduction using principal components analysis produced two components: sugar uptake and inflammatory EE. Tertiles were used to define EE severity as: lower, middle, and upper. The main outcome, combined prediabetes and diabetes, was defined as 2-hour oral glucose tolerance test (OGTT) glucose ≥7.8 mmol/L. Lower homeostatic model assessment (HOMA)-β and insulinogenic index, higher HOMA-insulin resistance (HOMA-IR), and lower Matsuda index were secondary outcomes. Logistic regression assessed the associations and HIV and BMI groups were tested as effect modifiers.

**Results:**

A total of 612 participants were included. The mean (±SD) age was 42.0 (±11.6) years and 57.2% (350) were females. Eighty (13%) were underweight, 367 (60%) normal weight, 165 (27%) overweight, and 357 (58%) were HIV-infected. We found no overall association of EE on the main outcome, but BMI and HIV modified the associations. Compared to lower tertile of sugar uptake EE, the upper tertile was associated with marginally significant higher odds of prediabetes and diabetes (OR=2.1 (95% CI: 0.9, 4.9; *P* = 0.06)) and marginally significant higher HOMA-IR (OR=2.6 (1.0, 6.8; *P* = 0.06*))* among overweight and obese participants. Similarly, compared to the lower tertile, the upper tertile of inflammatory EE was associated with higher odds of prediabetes and diabetes (OR=2.1 (1.1, 4.1; *P* = 0.03)) among HIV-uninfected participants, whereas among HIV-infected participants those in the middle tertile compared to those in the lower tertile had higher odds of lower Matsuda index (OR=2.3 (1.1, 4.7; *P* = 0.03)).

**Conclusions:**

EE may increase the risk of prediabetes and diabetes among those who are overweight and in individuals who are not HIV-infected. Longitudinal studies on the role of EE on diabetes are needed to confirm these results to provide the basis for developing and testing novel interventions to combat diabetes in Africa.

## Introduction

Diabetes is one of the major non-communicable diseases (NCDs) globally which is projected to affect 1.3 billion people by 2050 up from 529 million people in 2019 [1]. Diabetes is increasing more rapidly and the proportion of mortality attributable to diabetes is higher in low-and middle-income countries (LMICs) than in the high-income countries [2]. In LMICs, diabetes may occur in relatively young individuals compromising their health and reducing their productivity at the prime of their lives. In addition to diabetes-related complications, diabetes is a risk factor for hypertension, heart and kidney diseases and thus it may elevate the risk of premature mortality in affected individuals [3].

The increasing burden of diabetes in LMICs is probably related to multiple factors which may mediate persistent elevated plasma glucose level by either reducing secretion of insulin and/or compromising insulin action on the target cells [4] including physical inactivity, unhealthy diet, harmful use of alcohol and tobacco [5]. In addition, in both HIV-infected and HIV-uninfected populations, low grade inflammation secondary to inflammatory disorders like environmental enteropathy (EE) may increase the risk of NCDs including diabetes [6], but to date, EE has been little studied in relation to diabetes in Sub-Saharan Africa (SSA).

EE is a gut condition characterized by intestinal inflammation, abnormal changes in the morphology of intestinal mucosa and increased intestinal permeability and reduced absorption; it is caused by enteric infections and micronutrient deficiencies [7] which are common in settings with poor sanitation such as in many countries in SSA [8]. EE may lead to leaky gut resulting in translocation of microbes and endotoxins to the systemic circulation culminating in systemic inflammation and immune activation which may increase insulin resistance and diabetes risk [9]. In addition, translocation of bacterial products may lead to complex immunological reactions in the peritoneal organs and mesenteric fat [10] resulting in adipose tissue hyperplasia and hypertrophy, and visceral obesity [11]. Increased visceral adipose tissue may enhance secretion of excess free fatty acids and adipokines, including tumour necrosis factor (TNF)-α which increase hepatic and muscle insulin resistance [12,13] resulting in a higher diabetes risk. Also, EE may lead to loss of functions of incretin secreting intestinal neuroendocrine L cells leading to insulin insufficiency [14].

EE may contribute to a higher diabetes risk due to insulin resistance and reduced insulin secretion [15]. However, to date there have not been well-designed studies to elucidate the association between EE and diabetes among African populations. This hampers efforts to evaluate promising interventions in the management of diabetes in SSA populations including those infected with HIV. In this study we investigated if EE is associated with higher prediabetes and diabetes risk. We also explored if elevated risk could be explained by increased insulin resistance secondary to elevated systemic inflammation due to translocation of intestinal bacterial products to systemic circulation or reduced β-cell function.

## Methods

### Study design and setting

This analysis was part of the Role of Environmental Enteropathy on HIV-Associated Diabetes (REEHAD) study, a cross-sectional study investigating the association between EE and diabetes in Mwanza, Tanzania (clinicaltrials.gov NCT03713502). REEHAD is nested within the Chronic Infections, Co-morbidities and Diabetes in Africa (CICADA), a prospective cohort study investigating the burden of, and risk factors for diabetes and other NCDs among Tanzanian individuals with and without HIV [16]. The REEHAD study enrolled participants between 1^st^ May 2019 and 31^st^ March 2021.

### Data collection

#### Demography, socioeconomic status and NCDs risk factors.

Data on demography, smoking, alcohol intake, consumption of vegetables and fruits as well as physical activity were collected using WHO STEPs questionnaire [17]. Smoking history was classified as never, past and current smoking, while alcohol intake was classified into never taken or ever taken groups. Intake of ≥5servings per day (400g) of vegetable and fruits was deemed adequate according to WHO [4,18]. The WHO Global Physical Activity Questionnaire (GPAQ) was used to collect reported data on the level of physical activity [8]. Total physical activity was computed in metabolic equivalents of tasks (MET) in minutes per week and categorized as adequate if MET was ≥ 600 as recommended by WHO [19]. 

#### Anthropometry and body composition.

While barefoot and with minimal clothing, weight of the participant was determined to the nearest 0.1 kg using a digital scale (Seca, Germany), and height measured to the nearest 0.1 cm using a stadiometer fixed to the wall (Seca, Germany). Anthropometric measurements were taken in triplicate and medians were used for analysis. Based on weight and height measurements, body mass index (BMI) was calculated as mass (kg)/height (m)^2^ and classified as underweight (BMI < 18.5 kg/m^2^), normal weight (BMI 18.5 to 24.9 kg/m^2^) and overweight or obese (BMI ≥ 25 kg/m^2^) [16].

#### Diabetes mellitus, β-cell function, insulin resistance and HIV assessment.

Pre-diabetes and diabetes was the primary outcome whereas β-cell dysfunction and insulin resistance were secondary outcomes. To assess these outcomes, participants were contacted one day prior to the clinic visit and instructed to come fasting. On arrival in the clinic, they provided venous blood for fasting glucose measurement (Hemocue 201 RT, Angelholm, Sweden). Thereafter they were given 82.5g of dextrose monohydrate (equivalent to 75g of anhydrous glucose) diluted in 250 ml of drinking water to drink within 5 minutes for oral glucose tolerance test (OGTT), and blood samples for glucose were collected after 30 minutes and 120 minutes. Venous blood samples drawn at the same time as those for glucose assessment were separated into plasma for insulin (fasting, 30 minutes and 120 minutes) assessment. According to WHO guidelines, participants whose 120 minutes OGTT glucose level was ≥ 7.8 to < 11.1 mmol/L were classified as having impaired glucose tolerance (IGT), in this study termed as prediabetes (PD), and those with glucose level of ≥ 11.1 mmol/L were classified as having diabetes [10,11]. Combined prediabetes and diabetes was the main outcome measure as described above. Using insulin and OGTT data, we computed homeostatic model assessment-β (HOMA-β) and insulinogenic index (IGI) as markers of β-cell function, and HOMA-insulin resistance (HOMA-IR) and Matsuda index as markers of insulin resistance [12,13,20] (S1 Table**).** Using Liu’s method [21], these markers were dichotomized using optimal cut-off-points to indicate status of β-cell dysfunction and insulin resistance. The cut-off points were derived from a CICADA cohort in northwestern Tanzania with a sample of 1890 participants [20]. β-cell dysfunction was defined as IGI < 0.71 (mU/L)/(mmol/L) or HOMA-β index <38.3 (mU/L)/(mmol/L) [12–14,22]. Insulin resistance was defined as HOMA-IR index >1.9 (mU/L)/(mmol/L) or Matsuda index <7.2 (mU/L)/(mmol/L) [13,14,21,23,24]. HIV testing was done using two rapid antibody tests, i.e., SD Bioline HIV- 1/2 3.0 (standard diagnostics Inc, South Korea) and the Uni-Gold test (Trinity Biotech, Wicklow, Ireland). Discordant samples were tested using Uniform II vironostika-HIV Ag/Ab Micro-Elisa system (Biomerieuxbv, The Netherlands).

#### Assessment of EE.

To assess EE and potential pathways to prediabetes and diabetes, we collected stool and plasma samples to measure levels of intestinal inflammatory marker, i.e., fecal myeloperoxidase (MPO) (GenWay, Biotech, Santiago,CA, USA), serum lipopolysaccharide binding protein (LBP) as a marker of bacterial translocation (Hycult Biotech, Uden, Netherlands) and C-reactive protein (CRP) as a marker of systemic inflammation (COBAS-Roche, Basel, Switzerland). In addition, a day after an OGTT test was performed, an oral test solution containing 0.2 g of 3-O-methyl-D-glucose, 0.5 g of D-xylose, 1 g of L-rhamnose and 5 g of lactulose dissolved in 100mls of distilled water was administered to participants to assess intestinal absorption capacity and permeability after an overnight fasting and voiding of bladder [25,26]. Urine was collected for 3 hrs starting after administration of the test solution and the sugar content of the urine was analysed using ultra-performance liquid chromatography-mass spectrometry [27] at Specialist Bioanalytical Services Limited laboratory at the University of East London, UK. The amounts of sugars recovered were expressed as a percent recovery of the amount administered. 3-O-methyl-D-glucose is absorbed by active carrier-mediated process, D-xylose by passive carrier-mediated and rhamnose is absorbed transcellularly. Thus, EE-associated intestinal dysfunction may decrease absorption capacity of these sugars leading to reduced urine recovery [25,28]. In contrast, lactulose is absorbed through damaged tight junctions between enterocytes, therefore disruption of tight junctions associated with EE might lead to increased translocation, resulting in increased urine recovery [25,28].

#### Sample size and power considerations.

We hypothesized that EE would be associated with combined prediabetes and diabetes. Assuming the prevalence of the outcome among participants without or with minimal exposure to EE was about 20% [16] and equal size of the EE-exposed and unexposed groups, we would need a minimum of 374 participants to demonstrate the association with odds ratio of 2, with 80% power at 5% significant level.

### Data management and analysis

Data were entered into CSPro and analyzed in STATA 15. Background characteristics of the study participants including age, sex, alcohol drinking, smoking, physical activity, fruit and vegetable intake, BMI, HIV status as well as markers of EE and glucose metabolism were presented as percentages or means (SD) or median (IQR). Group differences of continuous variables were assessed using student t-test. We developed a composite index of EE, using principal component analysis (PCA), an analytical method used to generate indices requiring data reduction approaches [29] which has been used previously to investigate EE in LMICs [15]. Components for subsequent analysis were retained if they had an Eigen value of ≥ 1 [29] and their scores were divided into terciles for analysis with higher tertiles indicating increased severity of EE.

Initially, scatter graphs were used to explore plasma glucose during OGTT according to the severity of EE. Then the association between combined prediabetes and diabetes, β-cell dysfunction (i.e., HOMA-β and IGI), and insulin resistance (i.e., HOMA-IR and Matsuda index) with EE were computed using logistic regression. We initially conducted crude analyses and final models were adjusted for age, sex and physical activity. We did not adjust for risk/protective factors for dysglycaemia like fruits and vegetable intake, BMI, and HIV because these might be on the causal pathways between exposure and outcomes. However, we tested if BMI categories (i.e., underweight, normal weight and overweight/obese) and HIV modified the association between exposure and outcomes because previous research had suggested that these might be effect modifiers when evaluating predictors of prediabetes and diabetes [30,31]. When the test for interactions for effect modification was significant, main results were also presented by the strata of these modifiers. To explore potential pathways of the association between EE and a combined prediabetes and diabetes, we assessed associations between inflammatory/translocation markers with intestinal permeability and absorption markers (i.e., MPO, LBP, CRP, Lactulose, 3-O-methyl-D-glucose, D-xylose, and L-rhamnose) using linear regression and associations among permeability and absorption markers using spearman correlation. In regression analysis, if variables were non-normal, we log-transformed them before analysis. *P* value <0.05 was considered statistically significant.

### Ethical considerations

Ethics approval for investigations additional to the already approved CICADA study was obtained from the Medical Research Coordinating Committee (MRCC) based at NIMR and the London School of Hygiene and Tropical Medicine. Participants were enrolled in the study after they provided written informed consent and were free to leave the study any time after they had been enrolled. Data collected were kept confidential and access was restricted to study personnel responsible for data management, analysis and reporting who accessed study database using passwords. Participants who were recruited in the study and found to have diabetes or other diseases were referred to an appropriate health care facility for management. Costs incurred by participants due to participating in this study (e.g., travel costs) were reimbursed by the study. The study was conducted according to ethics principles enshrined in the Declaration of Helsink.

## Results

Of 1174 participants recruited in the REEHAD study, 612 (52.1%) had data on glucose and EE and were included in the analysis. Of those missing, 546 (46.5%) had no EE data, 8 (0.7%) had no glucose data and 8 (0.7%) missed both glucose and EE data. The mean (±SD) age was 42.0 (±11.6) years and 350 (57.2%) were females (Table 1). About 6% (36) were current smokers while 18.1% (111) smoked in the past, 69.3% (424) ever consumed alcohol and 58.3% (357) were HIV-infected. Only 75 (12.8%) had adequate intake of vegetables and fruits but most participants 552 (90.2%) were physically active. The median fecal MPO was 4.9 µg/ml (IQR:2.8, 8.7; normal range <2 µg/ml) and 85.6% (524) had elevated MPO. Median serum LBP was 7.8 µg/ml (IQR: 4.4, 12.4; normal range 5–15 µg/ml [32]) and 16.7% (102) had elevated LBP. The percentage of sugar recovery in urine ranged from 0.1% for lactulose to 15.9% for 3-O-methyl-D-glucose. About 28% (182) had prediabetes and diabetes and the proportion with β-cell dysfunction was 32.1% (143) by IGI and 47.1% (219) by HOMA-β. The prevalence of insulin resistance was 37.9% (176) by HOMA-IR but 49.6% (190) by Matsuda.

**Table 1:** Background characteristics of 612 participants included in the analysis.

Age (years)	42.0 (±11.6)
Female sex	350 (57.2)
Smoking	
Never	465 (76.0)
Ever	111 (18.1)
Current	36 (5.9)
Ever taken alcohol	424 (69.3)
Intake of ≥5 servings of fruits and vegetables	78 (12.8)
Physically active	552 (90.2)
Body mass index (BMI) (kg/m^2^)	22.9 (±4.7)
Underweight (BMI < 18.5 kg/m^2^)	80 (13.0)
Normal weight (BMI 18.5–24.9 kg/m^2^)	367 (60.0)
Overweight and obese (BMI ≥ 25 kg/m^2^)	165 (27.0)
Myeloperoxidase (µg/ml)	4.9 (2.8, 8.7)
Lipopolysaccharide binding protein (µg/ml)	7.8 (4.4,12.4)
Percentage of sugar recovered in urine	
D-Xylose	13.2 (7.6, 23.6)
3-O-methyl-D-glucose	15.9 (8.9, 25.3)
L-Rhamnose	2.4 (0.9, 4.9)
Lactulose	0.1 (0.06, 0.2)
Insulinogenic index (mU/L)/(mg/dL)	1.1 (0.6, 1.9)
β-cell dysfunction	143 (32.1)
HOMA-β (mU/L)/(mmol/L)	39.0 (24.3, 64.9)
β-cell dysfunction,	219 (47.1)
HOMA-IR (mU/L)/(mmol/L)	1.5 (0.9, 2.4)
Insulin resistance	176 (37.9)
Matsuda index (mU/L)/(mmol/L)	7.2 (4.8, 10.7)
Insulin resistance	190 (49.6)
Prediabetes and Diabetes	182 (29.7)
HIV-infected	357 (58.3)

Data are mean (±SD), n (%) and median (IQR).

HOMA-β, Homeostatic model assessment-β; HOMA-IR, Homeostatic model assessment-Insulin Resistance.

### EE patterns

In the PCA of 6 enteropathy markers, the first two components, with eigenvalues of 2.5 and 1.0, explained 59% of the total variation and were retained for further analysis (S2 Table). Kaiser-Meyer-Olkin’s measure was 0.8, indicating sampling adequacy. Variables with factor loadings ≥ |0.25| were used to name the patterns in each component. The first component had high positive loadings for intestinal permeability and absorption capacity sugar markers of lactulose, D-xylose, L-rhamnose, and 3-O-methyl-D-glucose, therefore it was designated sugar uptake EE. The second component was positively loaded with inflammatory markers of MPO and LBP and was designated as inflammatory-driven EE.

### Association of EE and markers of glucose metabolism

**Fig 1:**
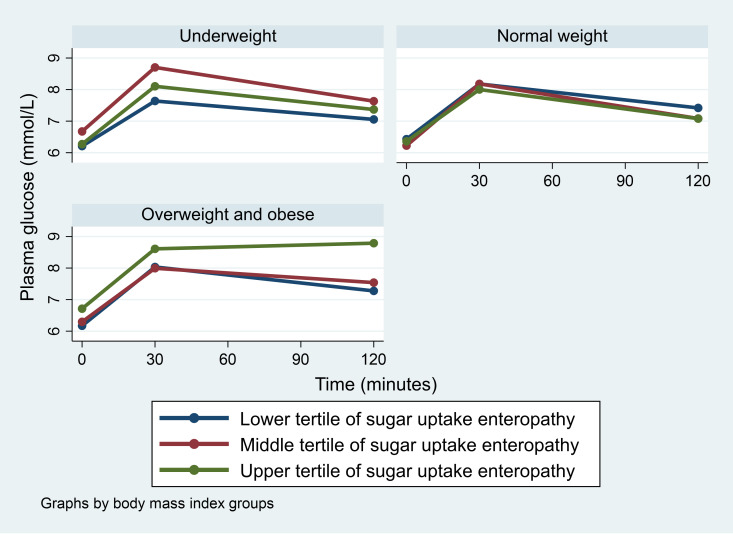
Glucose level during oral glucose tolerance test among tertiles of sugar uptake enteropathy by body mass index groups.

**Fig 2:**
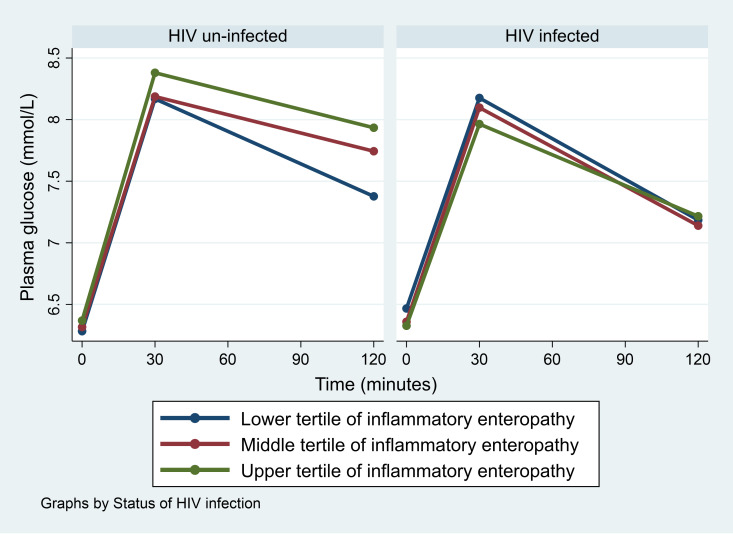
Glucose level during oral glucose tolerance test among tertiles of inflammatory enteropathy by status of HIV infection.

We found no overall association of EE on combined prediabetes and diabetes (Table 2), but BMI modified associations with sugar uptake EE and HIV with inflammatory EE (*P = *0.03 and *P = *0.05, respectively) (Table 3). In analyses of EE and glucose levels at 120 minutes, we found among overweight and obese participants that those in the upper tertile of sugar uptake EE had higher glucose level compared to those in the lower tertile (*P* = 0.01) (Fig 1). Similarly, HIV-uninfected participants in the upper tertile of inflammatory EE had higher level of glucose compared to those in the lower tertile, although this did not reach statistical significance (*P = *0.10) (Fig 2). In addition, we found that lactulose was positively correlated with markers of intestinal absorption capacity (i.e., D-xylose, L-rhamnose, 3-O-methyl-D-glucose) (*P < 0.001, all)* (Table 4 and S1 Fig).

**Table 2:** Association of environmental enteropathy with prediabetes and diabetes.

Sugar uptake environmental enteropathy						Inflammatory environmental enteropathy				
	N	OR (95%CI)^1^	*P*	OR (95% CI)^2^	*P*	N	OR (95%CI)^1^	*P*	OR (95% CI)^2^	*P*
Lower tertile	203	Reference		Reference		204	Reference		Reference	
Middle tertile	204	0.9 (0.6, 1.4)	0.69	0.9 (0.6, 1.4)	0.67	204	1.2 (0.8, 1.9)	0.32	1.2 (0.8, 1.8)	0.45
Upper tertile	205	1.1 (0.7, 1.6)	0.77	1.1 (0.7, 1.7)	0.62	204	1.4 (0.9, 2.1)	0.13	1.3 (0.9, 2.0)	0.20

^1^Unadjusted logistic regression model ^2^Logistic regression model adjusted for age, sex and physical activity.

**Table 3:** Association of sugar uptake and inflammatory environmental enteropathy with prediabetes and diabetes according to effect modification strata.

Sugar uptake environmental enteropathy by body mass index categories															
Environmental enteropathy tertiles	Underweight (n = 80)				Normal weight (n = 367)				Overweight and obese (n = 165)						*P* ^*3*^
	OR (95%CI)^1^	*P*	OR (95% CI)^2^	*P*	OR (95%CI)^1^	*P*	OR (95% CI)^2^	*P*	OR (95%CI)^1^		*P*		OR (95% CI)^2^	*P*	
Lower tertile	Reference		Reference		Reference		Reference		Reference				Reference		0.03
Middle tertile	0.6 (0.1, 2.3)	0.46	0.6 (0.1, 2.3)	0.42	0.9 (0.5, 1.6)	0.81	0.9 (0.5, 1.6)	0.80	0.9 (0.4, 2.2)		0.99		0.9 (0.4, 2.2)	0.93	
Upper tertile	1.6 (0.4, 5.4)	0.45	1.6 (0.4, 5.7)	0.48	0.7 (0.4, 1.2)	0.18	0.7 (0.4, 1.3)	0.28	2.1 (1.0, 4.7)		0.04		2.1 (0.9, 4.6)	0.06	
Inflammatory environmental enteropathy by HIV status															
Environmental enteropathy tertiles	HIV-uninfected (n = 255)				HIV-infected (n = 357)				*P* ^*4*^						
	OR (95%CI)^1^	*P*	OR (95% CI)^2^	*P*	OR (95%CI)^1^	*P*	OR (95% CI)^2^	*P*							
Lower tertile	Reference		Reference		Reference		Reference		0.05						
Middle tertile	1.3 (0.7, 2.5)	0.37	1.3 (0.7, 2.5)	0.42	1.2 (0.7, 2.1)	0.58	1.1 (0.6, 2.0)	0.72							
Upper tertile	2.2 (1.1, 4.1)	0.02	2.1 (1.1, 4.1)	0.03	1.0 (0.6, 1.9)	0.89	1.0 (0.6, 1.8)	0.98							

^1^Unadjusted logistic regression model ^2^Logistic regression model adjusted for age, sex, and physical activity ^*3*^Test for interaction between sugar uptake environmental enteropathy with body mass index categories ^*4*^Test for interaction between inflammatory environmental enteropathy with HIV status.

**Table 4:** Spearman correlations among intestinal absorption and permeability sugars^1.^

	Lactulose	Xylose	Rhamnose
Xylose	rs = 0.63 *P < 0.0001*		
Rhamnose	rs = 0.54 *P < 0.0001*	rs = 0.77 *P < 0.0001*	
3-O-methyl-D-glucose	rs = 0.62 *P < 0.0001*	rs = 0.81 *P < 0.0001*	rs = 0.74 *P < 0.0001*

rs = Spearman correlation coefficient ^1^Correlations were calculated using percentage of sugars recovered in urine.

In a model adjusted for age, sex and physical activity, upper tertile of sugar uptake EE was associated with marginally significant higher odds of prediabetes and diabetes (OR=2.1 (95% CI: 0.9, 4.9; *P* = 0.06)) (Table 3) and corresponding marginally significant higher odds of higher HOMA-IR (OR=2.6 (1.0, 6.8; *P* = 0.06*))* among overweight and obese participants (Table 5). Similarly, compared to the lower tertile of inflammatory EE, the upper tertile was associated with higher odds of prediabetes and diabetes (OR=2.1 (1.1, 4.1; *P* = 0.03)) among HIV-uninfected participants (Table 3), whereas among HIV-infected participants those in the middle tertile compared to those in the lower tertile of inflammatory EE had higher odds of lower Matsuda index (OR=2.3 (1.1, 4.7; *P* = 0.03)) (Table 6).

**Table 5:** Association of intestinal sugar uptake enteropathy with markers of insulin resistance and β-cell dysfunction by nutritional status.

	Lower insulinogenic index				Lower HOMA-β				Higher HOMA-IR				Lower Matsuda Index			
	OR (95%CI)^1^	*P*	OR (95% CI)^2^	*P*	OR (95%CI) ^1^	*P*	OR (95%CI) ^2^	*P*	OR (95%CI) ^1^	*P*	OR (95% CI) ^2^	*P*	OR (95%CI)^1^	*P*	OR (95% CI) ^2^	*P*
Underweight (N = 80)																
Lower tertile	Reference		Reference		Reference		Reference		Reference		Reference		Reference		Reference	
Middle tertile	1.6 (0.4, 6.1)	0.52	1.7 (0.4, 7.1)	0.47	0.3 (0.1, 1.5)	0.14	0.3 (0.1, 1.5)	0.14	5.2 (0.9, 30.2)	0.07	4.5 (0.7, 28.5)	0.11	35.1 (1.8, 693.4)	0.01	42.2 (1.8, 987.4)	0.02
Upper tertile	2.7 (0.7, 11.2)	0.16	2.7 (0.6, 11.4)	0.18	0.3 (0.1, 1.7)	0.19	0.3 (0.1, 1.8)	0.21	2.4 (0.3, 16.4)	0.38	2.5 (0.3, 18.4)	0.38	17.9 (0.9, 362.5)	0.06	14.2 (0.7, 281.2)	0.08
Normal weight (N = 367)																
Lower tertile	Reference		Reference		Reference		Reference		Reference		Reference		Reference		Reference	
Middle tertile	0.7 (0.4, 1.5)	0.40	0.8 (0.4, 1.5)	0.46	1.0 (0.6, 1.8)	0.97	0.9 (0.5, 1.7)	0.79	0.8 (0.4, 16)	0.64	1.0 (0.5, 1.9)	0.93	1.0 (0.5, 2.0)	0.90	1.2 (0.6, 2.3)	0.68
Upper tertile	1.2 (0.7, 2.2)	0.49	1.4 (0.7, 2.6)	0.31	1.2 (0.7, 2.0)	0.54	1.2 (0.7, 2.2)	0.49	0.9 (0.5, 1.7)	0.81	0.9 (0.5, 1.8)	0.81	0.8 (0.4, 1.4)	0.37	0.7 (0.4, 1.5)	0.39
Overweight and obesity (N = 165)																
Lower tertile	Reference		Reference		Reference		Reference				Reference		Reference		Reference	
Middle tertile	2.0 (0.7, 6.2)	0.22	1.7 (0.6, 5.5)	0.34	0.9 (0.3, 2.6)	0.86	0.9 (0.3, 2.5)	0.80	1.2 (0.5, 3.0)	0.63	1.4 (0.6, 3.6)	0.45	1.4 (0.4, 4.2)	0.59	1.4 (0.4, 4.4)	0.59
Upper tertile	2.4 (0.8, 7.1)	0.12	2.1 (0.7, 6.4)	0.21	0.9 (0.3, 2.4)	0.78	0.8 (0.3, 2.3)	0.63	2.1 (0.8,5.2)	0.13	2.6 (1.0, 6.8)	0.06	3.0 (0.8, 11.2)	0.10	2.9 (0.7, 11.7)	0.12

^1^Unadjusted logistic regression model ^2^Logistic regression model adjusted for age, sex and physical activity HOMA-β, Homeostatic Model Assessment-β HOMA-IR, HOMA-Insulin Resistance.

**Table 6:** Association of inflammatory environmental enteropathy with markers of insulin resistance and β-cell dysfunction by HIV status.

	Lower insulinogenic index				Lower HOMA-β				Higher HOMA-IR				Lower Matsuda Index			
Classes of inflammatory environmental enteropathy^1^	OR (95%CI)^1^	*P*	OR (95% CI)^2^	*P*	OR (95%CI)^1^	*P*	OR (95%CI) ^2^	*P*	OR (95%CI) ^1^	*P*	OR (95% CI)^2^	*P*	OR (95%CI)^1^	*P*	OR (95% CI)^2^	*P*
HIV-uninfected (N = 255)																
Lower tertile	Reference		Reference		Reference		Reference		Reference		Reference		Reference		Reference	
Middle tertile	1.7 (0.8, 3.6)	0.15	1.8 (0.8, 3.8)	0.14	0.5 (0.2, 0.9)	0.04	0.4 (0.2, 0.9)	0.02	0.7 (0.4, 1.4)	0.36	0.7 (0.4, 1.5)	0.38	1.3 (0.6, 2.7)	0.52	1.2 (0.6, 2.7)	0.60
Upper tertile	0.9 (0.4, 2.1)	0.93	0.9 (0.4, 2.1)	0.82	0.9 (0.4, 1.8)	0.76	1.0 (0.5, 2.1)	0.93	0.7 (0.4, 1.4)	0.37	0.6 (0.3, 1.3)	0.19	1.2 (0.6, 2.5)	0.68	0.9 (0.4, 2.2)	0.96
HIV-infected (N = 357)																
Lower tertile	Reference		Reference		Reference		Reference		Reference		Reference		Reference		Reference	
Middle tertile	1.1 (0.6, 2.1)	0.74	0.9 (0.5, 1.9)	0.91	1.1 (0.6, 2.1)	0.64	1.2 (0.6, 2.1)	0.60	1.0 (0.5, 1.9)	0.98	1.0 (0.5, 1.9)	0.99	1.9 (1.0, 3.9)	0.05	2.3 (1.1, 4.7)	0.03
Upper tertile	1.0 (0.5, 1.9)	0.91	0.9 (0.5, 2.0)	0.94	1.1 (0.6, 2.0)	0.70	1.2 (0.7, 2.3)	0.47	1.5 (0.8, 2.8)	0.17	1.4 (0.7, 2.6)	0.29	1.8 (0.9, 3.5)	0.07	1.7 (0.8, 3.3)	0.14

^1^Unadjusted logistic regression model ^2^Logistic regression model adjusted for age, sex and physical activity HOMA-β, Homeostatic Model Assessment-β HOMA-IR, HOMA-Insulin Resistance.

To understand potential pathways to study outcomes, we investigated relationships among EE markers and found LBP was not associated with either MPO or CRP, but MPO was positively associated with CRP in both HIV-uninfected (β = 0.20 (0.03, 0.37; *P* = 0.02)) and HIV-infected participants (β = 0.23(0.05, 0.43; *P* = 0.01)) (S3 Table). We found there was associations between MPO and LBP with D-xylose, L-rhamnose, and 3-O-methyl-D-glucose and lactulose among the BMI groups, however, these were not statistically significant (S4 Table).

## Discussion

In this study among adults who were HIV-infected and those without HIV, we observed high levels of MPO and LBP and amounts of lactulose recovered in urine similar to those found in populations confirmed to have EE [25] indicating that our study population had cardinal markers of EE including intestinal inflammation and increased intestinal permeability. The hypothesis in this study was that EE may increase the risk of prediabetes and diabetes by increasing insulin resistance or due to decreased insulin secretion. We successfully used PCA to obtain meaningful and distinct components of EE, i.e., sugar uptake EE and inflammatory EE. Overall, we found no associations of EE with glucose metabolism markers. However, in sub-group analyses, we found that among overweight and obese participants, sugar uptake EE was associated with higher prediabetes and diabetes risk whereas among HIV-uninfected participants, inflammatory EE doubled the risk of pre-diabetes and diabetes.

EE is common in areas with poor sanitation and hygiene practices as in most poor regions of SSA. Although it can lead to elevated inflammation and increase the risk of cardiometabolic diseases, there are very limited studies exploring these associations. Among Peruvian infants who were followed at 3–5 years, EE-associated lower intestinal surface area and inflammation were associated with elevated blood pressure, altered adipokines, apolipoprotein, and cytokines during follow-up suggesting that early EE might increase the risk of later metabolic syndrome [6,33]. A Mexican study found that MPO was associated with insulin resistance and inflammation in overweight first degree relatives of diabetes patients [34] and a Brazilian study among the general population found that MPO was a predictor of markers of cardiometabolic diseases including hypertension and body mass index [35]. These studies did not assess the association with diabetes; therefore, our results are novel, and contribute to the accumulating evidence on the associations of EE and cardiometabolic diseases.

### Sugar uptake EE and risk of prediabetes and diabetes

In this study we found that among overweight and obese participants, upper tertile of sugar uptake EE was associated with prediabetes and diabetes. We think this could be due to at least two reasons. First, overweight and obese individuals are known to have high level of systemic inflammation compared to normal weight individuals [36] as such increased permeability associated with high sugar permeation could have further increased systemic inflammation and insulin resistance following translocation of endotoxins to systemic circulation [37,38]. However, we found no evidence of association between markers of intestinal permeability and absorption capacity or LBP with CRP suggesting that other mechanisms might explain this association better or markers of intestinal barrier dysfunction assessed were not good predictors of systemic inflammation. Secondly, the observed association could be due to reverse causality. Recent studies have suggested that hyperglycaemia may induce intestinal barrier dysfunction and elevate risk of translocation of enteric infections to the systemic circulation [39,40]; thus, it is possible that dysglycaemia among overweight and obese participants led to increased intestinal permeability and not the reverse. Further studies to understand intestinal permeability and glycaemia dynamics will provide evidence base for developing interventions to combat the burden of diabetes in SSA.

### Inflammatory EE and risk of prediabetes and diabetes

Our results suggest that Inflammatory EE was associated with higher risk of prediabetes and diabetes among HIV-uninfected but not HIV-infected. In PCA, we found that inflammatory EE was driven by MPO, which is produced by neutrophils and is an inflammatory marker and LBP which is a marker of bacterial products translocation to systemic circulation. MPO was not associated with LBP but was positively associated with CRP suggesting that the effect of inflammatory enteropathy on prediabetes and diabetes was likely explained by independent effect of MPO on systemic inflammation. Therefore, contrary to our hypothesis, the effect of MPO was not mediated by LBP similar to a Japanese study among diabetes patients which reported LBP was not different among patients with or without bacteraemia [41]. It is possible that the association of LBP with prediabetes and diabetes was mediated by other markers which we did not assess, but further studies are needed to explore these mechanisms.

Unlike HIV-uninfected, we found that inflammatory EE was not associated with prediabetes and diabetes among HIV-infected, although there were indications that it was associated with higher insulin resistance. This is paradoxical since MPO was positively associated with CRP in both HIV-infected and HIV-uninfected participants. In addition, we found no evidence of compensatory improved β-cell function due to insulin resistance in HIV-infected which would tend to normalize glucose level. Therefore, we suggest that this may have been due to survival bias where most of those at higher risk of developing prediabetes and diabetes in HIV group either died or were lost to follow-up before data collection as we found in our other reports from this population among participants who were on ART for long period [16]. Future studies exploring these differences are mandated.

### Intestinal permeability and absorption markers

When exploring the correlations among sugars, we unexpectedly found that urine levels of D-xylose, L-rhamnose, and 3-O-methyl-D-glucose, markers of intestinal absorption capacity, had positive rather than negative correlation with lactulose which is a marker of permeability. This is difficult to explain, since it is expected in most cases of EE, lactulose recovery would increase whereas recovery of the other sugars would be reduced due to reduced absorption capacity. However, we think that in populations with severe EE, when permeation exceeds physiological absorption, extensive permeation of all molecules might occur through a leaky epithelium and generate positive correlations among molecules. Yet, over the time this leakiness may diminish as an adaptive strategy to limit severe effects of translocation of harmful bacterial products to systemic circulation [42]. However, the cross-sectional nature of this study limits efforts to predict if evolution of EE among adults would follow this trajectory. Future longitudinal studies are warranted to better understand intestinal permeability and absorption capacity relationships in adults.

### Strengths and limitations

This study had a large sample size therefore had high precision to estimate risks and large power to detect associations between groups for the primary outcomes. We collected exposure and outcome data using reference techniques. Due to funding limitations, we only analyzed few exposure variables, thus we may have failed to fully characterized EE which might have led to misclassification of exposure leading to reduced strength of association between EE and prediabetes and diabetes. However, this is unlikely given that we analyzed the most important markers for each stage of proposed causal pathway (i.e., from enteropathy to prediabetes and diabetes). This study recruited participants from CICADA cohort which is an observational study investigating risk factors of diabetes. If those who had diabetes at the beginning of the cohort died or were lost to follow-up before recruitment for the current study, this may have introduced survival bias particularly in HIV-infected participants. In addition, it is likely that we did not have enough power to detect all associations in sub-groups analyses defined by effect modifiers which could have led to some inconsistent findings. For example, we found middle tertile of inflammatory environmental enteropathy was associated with higher risk of lower Matsuda index among HIV-infected participants but similar association was not significant among those in the upper tertile. We suggest that a larger sample size among the tertiles would have led to more consistent results. Finally, this was a cross-sectional study and causality as well as directionality of the association cannot be confirmed.

## Conclusions

Inflammatory EE may increase the risk of prediabetes and diabetes among HIV-uninfected populations and sugar uptake EE may increase the risk of prediabetes and diabetes among overweight and obese participants. Thus, EE could be contributing to a higher burden of diabetes in SSA. We recommend longitudinal studies on the role of EE on diabetes to confirm these results and

mechanistic studies to better understand mechanisms linking intestinal inflammation and intestinal permeability with glucose dysglycaemia to provide data for developing novel interventions to combat diabetes in SSA.

## Supporting information

S1 Table Formulae for β-cell function and insulin resistance markers. (DOCX)

S2 Table Component characteristics and factor loadings of retained principal components. (DOCX)

S1 Fig Matrix graph of correlations among intestinal absorption and permeability sugars. (DOCX)

S3 Table Associations among myeloperoxidase, lipopolysaccharide binding protein and C-reactive protein by HIV status. (DOCX)

S4 Table Association of inflammatory markers with intestinal absorption capacity and permeability markers by body mass index groups. (DOCX)

S1 Text Inclusivity in global research questionnaire. (DOCX)
