# Supplementary material for: Association of environmental enteropathy with prediabetes and diabetes: A cross-sectional study among Tanzanian adults
Source: PLoS One. 2025 Jul 3;20(7):e0327166. doi: 10.1371/journal.pone.0327166 (PMC12225851; doi:10.1371/journal.pone.0327166)
Supplement: S1 Table — (DOCX) [file pone.0327166.s001.docx]

| **S1 Table: Formulae for β-cell function and insulin resistance markers** | | | |
| --- | --- | --- | --- |
| **Markers** | **Definition/formula^1^** | **Cut-off points for β-cell dysfunction and insulin resistance^2^** |  |
| **β-cell function** |  |  |  |
| Insulinogenic index | Change in insulin over change in glucose in first 30 minutes following OGTT. | <0.71 (mU/L)/(mmol/L) |  |
| HOMA-β | (20* Fasting blood insulin (FBI)/(Fasting plasma glucose (FPG)-3.5) | <38.3 (mU/L)/(mmol/L) |  |
| **Insulin resistance** |  |  |  |
| HOMA-IR | (FBI *FPG)/22.5 | >1.9 (mU/L)/(mmol/L) |  |
| Matsuda index | 1000/√FPG*FBI) (MPG)*(MPI) | <7.2 (mU/L)/(mmol/L) |  |
| ^1^Published definitions[27, 31-33, 53, 54] ^2^Published cut-off points using Liu’s method[29, 30, 55] HOMA-β, Homeostatic model assessment-β; HOMA-IR, HOMA-Insulin Resistance; FBG, Fasting plasma glucose, OGTT, Oral glucose tolerance test; MPG, mean plasma glucose at 0, 30 and 120 minutes; MPI, mean of plasma insulin at 0, 30, and 120 minutes. | | |  |
