# Supplementary material for: Association of environmental enteropathy with prediabetes and diabetes: A cross-sectional study among Tanzanian adults
Source: PLoS One. 2025 Jul 3;20(7):e0327166. doi: 10.1371/journal.pone.0327166 (PMC12225851; doi:10.1371/journal.pone.0327166)
Supplement: S2 Table — (DOCX) [file pone.0327166.s002.docx]

| S2 Table. Component characteristics and factor loadings of retained principal components | | |
| --- | --- | --- |
|  | First component | Second component |
| **Component characteristics** | | |
| Eigen value | 2.5 | 1.0 |
| Proportion of explained variance | 0.42 | 0.17 |
| Cumulative proportion of explained variance | 0.42 | 0.59 |
| **Factor loadings** | | |
| Myeloperoxidase | -0.04 | 0.69 |
| Lipopolysaccharide binding protein | -0.03 | 0.70 |
| D-xylose | 0.58 | 0.01 |
| L-rhamnose | 0.52 | -0.02 |
| 3-O-methyl-D-glucose | 0.56 | 0.03 |
| Lactulose | 0.27 | 0.17 |
