# Supplementary material for: Association of environmental enteropathy with prediabetes and diabetes: A cross-sectional study among Tanzanian adults
Source: PLoS One. 2025 Jul 3;20(7):e0327166. doi: 10.1371/journal.pone.0327166 (PMC12225851; doi:10.1371/journal.pone.0327166)
Supplement: S1 Fig — (DOCX) [file pone.0327166.s003.docx]

S1 Figure. Matrix graph of correlations among intestinal absorption and permeability sugars^1^

^1^All correlations among sugars, *P*<0.001
