# Supplementary material for: Association of environmental enteropathy with prediabetes and diabetes: A cross-sectional study among Tanzanian adults
Source: PLoS One. 2025 Jul 3;20(7):e0327166. doi: 10.1371/journal.pone.0327166 (PMC12225851; doi:10.1371/journal.pone.0327166)
Supplement: S3 Table — (DOCX) [file pone.0327166.s004.docx]

| S3 Table : Associations among myeloperoxidase, lipopolysaccharide binding protein and C-reactive protein by HIV status | | | | | | | | |
| --- | --- | --- | --- | --- | --- | --- | --- | --- |
|  | HIV-uninfected (N=255) | | | | HIV-infected (N=357) | | | |
|  | Log-transformed LBP | | Log-transformed CRP | | Log-transformed LBP | | Log-transformed CRP | |
|  | β (95%CI) | P | β (95%CI) | P | β (95%CI) | P | β (95%CI) | P |
| Log-transformed MPO | 0.07(-0.04, 0.19) | 0.22 | 0.20 (0.03, 0.37) | 0.02 | -0.03 (-0.14, 0.08) | 0.62 | 0.23 (0.05, 0.43) | 0.01 |
| Log-transformed LBP | - |  | 0.12 (-0.06, 0.30) | 0.19 | - |  | 0.09 (-0.08, 0.27) | 0.29 |
| MPO, Myeloperoxidase; LBP, Lipopolysaccharide binding protein; CRP, high sensitivity C-reactive protein; β, regression coefficient adjusted for age and sex. | | | | | | | | |
