# Supplementary material for: Association of environmental enteropathy with prediabetes and diabetes: A cross-sectional study among Tanzanian adults
Source: PLoS One. 2025 Jul 3;20(7):e0327166. doi: 10.1371/journal.pone.0327166 (PMC12225851; doi:10.1371/journal.pone.0327166)
Supplement: S4 Table — (DOCX) [file pone.0327166.s005.docx]

| S4 Table: Association of inflammatory markers with intestinal absorption capacity and permeability markers by body mass index groups | | | | | | | | |
| --- | --- | --- | --- | --- | --- | --- | --- | --- |
|  | Log-transformed Xylose | | Log-transformed Rhamnose | | Log-transformed Glucose | | Log-transformed Lactulose | |
|  | β (95%CI) | *P* | β (95%CI) | *P* | β (95%CI) | *P* | β (95%CI) | *P* |
| **Underweight (N=80)** |  |  |  |  |  |  |  |  |
| Log-transformed MPO | -0.1 (-0.38, 0.14) | 0.36 | -0.18 (-0.49, 0.12) | 0.25 | 0.03(-0.22, 0.29) | 0.79 | -0.02 (-0.26, 0.20) | 0.82 |
| Log-transformed LBP | 0.04 (-0.23, 0.32) | 0.75 | 0.09 (-0.23, 0.41) | 0.57 | -0.05 (-0.32, 0.22) | 0.72 | 0.12 (-0.11, 0.37) | 0.29 |
| Log-transformed CRP | 0.02 (-0.16, 0.21) | 0.79 | -0.09 (-0.32, 0.14) | 0.43 | 0.18 (-0.01, 0.38) | 0.06 | 0.09 (-0.09, 0.27) | 0.31 |
| **Normal weight (N=285)^a^** |  |  |  |  |  |  |  |  |
| Log-transformed MPO | 0.02 (-0.11, 0.08) | 0.75 | -0.03 (-0.17, 0.11) | 0.75 | 0.01 (-0.08, 0.10) | 0.80 | 0.10 (-0.01, 0.21) | 0.08 |
| Log-transformed LBP | -0.07 (-0.17, 0.03) | 0.17 | -0.02 (-0.16, 0.12) | 0.81 | -0.07 (-0.15, 0.02) | 0.14 | -0.10 (-0.22, 0.01) | 0.08 |
| Log-transformed CRP | 0.03 (-0.04, 0.10) | 0.38 | -0.04 (-0.14, 0.07) | 0.53 | -0.002 (-0.07, 0.07) | 0.95 | 0.07 (-0.02, 0.16) | 0.12 |
| **Overweight(N=165)** |  |  |  |  |  |  |  |  |
| Log-transformed MPO | -0.08 (-0.26, 0.10) | 0.40 | 0.08 (-0.13, 0.30) | 0.45 | -0.04(-0.20, 013) | 0.64 | 0.08 (-0.09, 0.25) | 0.37 |
| Log-transformed LBP | -0.06 (-0.24, 0.11) | 0.49 | 0.09 (-0.11, 0.31) | 0.38 | 0.06 (-0.10, 0.22) | 0.46 | -0.03 (-0.20, 0.13) | 0.69 |
| Log-transformed CRP | 0.01 (-0.13, 0.16) | 0.86 | -0.04 (-0.23, 0.15) | 0.65 | 0.01 (-0.13, 0.15) | 0.89 | 0.07 (-0.07, 0.21) | 0.31 |
| ^a^Data for 85 are missing; MPO, Myeloperoxidase; LBP, Lipopolysaccharide binding protein; CRP, high-sensitivity C-reactive protein; β, regression coefficient adjusted for age and sex. | | | | | | | | |
